# Bioactive Compounds and Their Neuroprotective Effects in Diabetic Complications

**DOI:** 10.3390/nu8080472

**Published:** 2016-07-30

**Authors:** Yoon Sin Oh

**Affiliations:** 1 Lee Gil Ya Cancer and Diabetes Institute, Gachon University, Incheon 406-723, Korea; 2 Gachon Medical Research Institute, Gil Hospital, Incheon 406-723, Korea; with62@gachon.ac.kr; Tel.: +82-32-899-6064; Fax: +82-32-899-6057

**Keywords:** flavonoids, vitamins, diabetes, neuroprotection

## Abstract

Hyperglycemia, hyperlipidemia and impaired insulin signaling during the development of diabetes can cause diabetic complications, such as diabetic neuropathy, resulting in significant morbidity and mortality. Although various therapeutics are available for the treatment of diabetic neuropathy, no absolute cure exists, and additional research is necessary to comprehensively understand the underlying pathophysiological pathways. A number of studies have demonstrated the potential health benefits of bioactive compounds, i.e., flavonoids and vitamins, which may be effective as supplementary treatments for diabetes and its complications. In this review, we highlight the most recent reports about the mechanisms of action of bioactive compounds (flavonoids and vitamins) possessing potential neuroprotective properties in diabetic conditions. Additional clinical studies are required to determine the appropriate dose and duration of bioactive compound supplementation for neuroprotection in diabetic patients.

## 1. Introduction

Diabetes, a complex metabolic disorder caused by insulin insufficiency and/or insulin dysfunction, is characterized by abnormal blood glucose and insulin levels. Type 1 diabetes is caused by cell-specific autoimmune destruction of the insulin-producing beta cells in the pancreas [1]. Type 2 diabetes is a result of the failure of beta cells to compensate for insulin resistance or selective loss of pancreatic beta cells due to toxic damage, leading to insulin insufficiency [2].

Uncontrolled hyperglycemia resulting from type 1 and type 2 diabetes may cause chronic tissue dysfunction and organ failure, such as atherosclerosis, retinopathy, nephropathy and neuropathy [3]. Diabetic complications are considered risk factors for morbidity and mortality in patients with diabetes.

**Figure 1:**
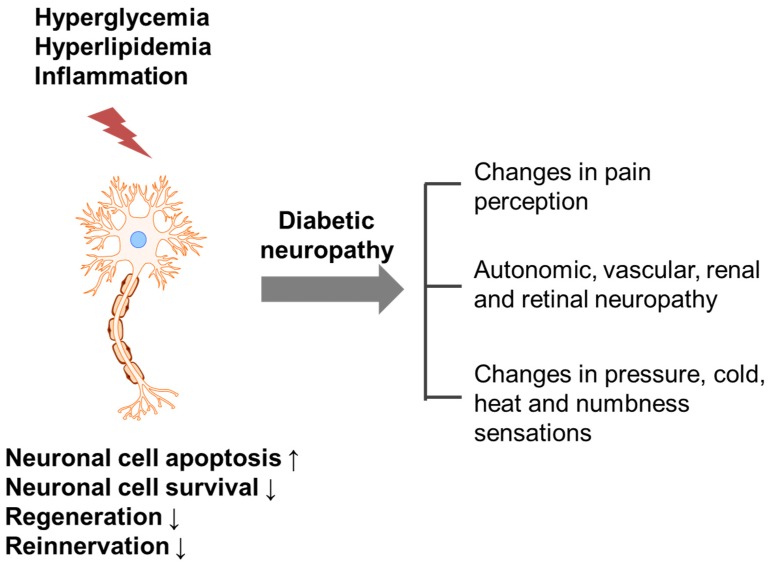
Possible trigger and mechanism underlying the development of diabetic neuropathy. Hyperglycemia, hyperlipidemia and inflammation induce nerve damage, which increases apoptosis and decreases cell survival, regeneration and reinnervation, resulting in diabetic neuropathy.

**Figure 2:**
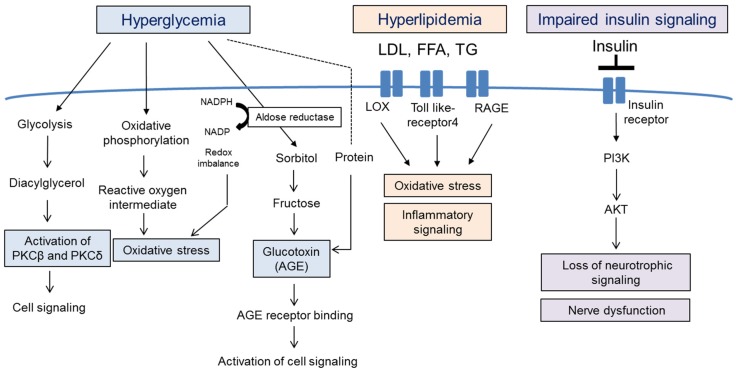
Pathophysiology of neuronal cell death and dysfunction during diabetes development. Uncontrolled hyperglycemia, hyperlipidemia and impaired insulin signaling occur during the development of diabetes and diabetic neuropathy. Hyperglycemia activates glycolysis, oxidative phosphorylation and aldose reductase pathways, resulting in the formation of oxidative stress, glycation end-products (AGE) and protein kinase c (PKC)-mediated cell signaling molecules. Elevated levels of LDL, FFA and TG activate oxidative stress, and impaired insulin signaling induces nerve dysfunction via inhibition of neurotrophic signaling. PKC, protein kinase C; AGE, advanced glycation end products; LDL, low-density lipoprotein; FFA, free fatty acid; TG, triglyceride; RAGE, receptor for advanced glycation end products; LOX, oxidized LDL receptor 1; PI3K, phosphatidylinositol 3-kinase.

Diabetic neuropathy refers to any condition that affects the normal activity of the nervous system during hyperglycemia, hyperlipidemia and inflammation. Damaged (apoptotic or non-innervated) neuronal cells can affect pain perception and sensation changes (Figure 1) [4]. It has been reported that 60%–70% of diabetic patients suffer from neuropathy; by the year 2030, approximately 236 million people will be affected worldwide [5,6]. Increased free radical formation and compromised antioxidant defense systems have been implicated in the development of diabetic neuropathy [7,8], but more complex pathophysiological pathways, such as hyperglycemia, hyperlipidemia and impaired insulin signaling, are also likely to be involved (Figure 2).

## 2. Causes of Diabetic Neuropathy and Related Pathophysiology

### 2.1. Hyperglycemia

Hyperglycemia is a major initiator of the pathophysiology of diabetes and leads to the development of neuropathy and neuropathic pain. Several theories have been proposed to explain how hyperglycemia can cause neuronal derangements. Hyperglycemia activates biochemical pathways, such as glycolysis, the aldose reductase pathway (polyol pathway) and oxidative phosphorylation, all of which result in the formation of reactive oxygen species (ROS), advanced glycation end-products (AGE) and protein kinase c (PKC)-mediated cell signaling molecules [9].

Excess glycolysis can lead to the overload of the mitochondrial electron transport chain and the generation of ROS [10]. Moreover, increased glycolysis can increase activation of the PKCβ and PKCδ pathways and alter gene expression of cytokines and growth factors. Increased activation of the polyol pathway reduces NADPH, leading to oxidative stress. Moreover, AGE production was shown to be enhanced via the attachment of reactive carbohydrate groups to proteins. AGEs bind to the AGE receptor (RAGE), initiating the inflammatory signaling pathway [11]. Therapeutic strategies for diabetic neuropathy include neutralization of specific glucotoxins and blocking of hyperglycemia-induced biochemical pathways, but both showed a lack of efficacy in human trials, and side effects and toxicity have been observed [12,13,14].

### 2.2. Hyperlipidemia

The occurrence rate of hyperlipidemia is high in type 2 diabetes patients, and lipid profiles are abnormal early on in the development of diabetic neuropathy, suggesting that hyperlipidemia may be involved in diabetic neuropathy. Indeed, increased levels of free fatty acids directly caused injury to neuronal cells [15], and oxidized low-density lipoproteins (LDLs) trigger signaling cascades that activate NADPH oxidase and induce oxidative stress via LDL receptors (LDL receptor LOX1, Toll-like receptor 4 and RAGE) [16,17]. Clinical trials on the use of lipid-lowering drugs for the treatment of diabetic neuropathy have been performed, yielding controversial results [18,19].

### 2.3. Impairment of Insulin Signaling

Insulin has neurotrophic properties, i.e., it promotes neuronal growth and neuronal survival [20]. Phosphatidylinositol-3-kinase/protein kinase B (PI3K/AKT) signaling is activated by neurotrophic signaling, and disruption of this pathway leads to nerve dysfunction, as evidenced by decreased sodium-potassium-ATPase activity, endothelial nitric oxide synthase (eNOS) activity and cerebral blood flow [21]. Moreover, insulin resistance affects neuronal repair mechanisms and decreases the levels of nerve growth factors [22]. Therefore, induction of brain neurotrophins, sodium-potassium-ATPase activity and nerve growth factor (NGF) could be beneficial for the treatment or prevention of diabetic neuropathy.

Many agents targeting different pathways have been studied and used for the treatment of diabetic neuropathy. Several medications for relief from nerve pain are available, but the treatment of diabetic neuropathy is difficult, as not all medications are effective in all patients, and most have side effects, such as swelling, dizziness and weight gain [23].

Bioactive compounds from foods are important sources of safe, specific and effective anti-neuropathic agents and may be useful in the development of safer alternatives to pharmaceuticals. Flavonoids are the most abundant polyphenolic compounds found in the human diet, and more than 5000 naturally-occurring flavonoids are currently known to be present in various plants. These compounds have several beneficial effects on human health [24]. Vitamins also function as antioxidants, which have diverse chemical structures and biochemical functions. Recent literature suggested that some bioactive compounds possess both neuroprotective and neurotrophic actions [25]; therefore, early treatment of peripheral neuropathy using phytochemical approaches may be an important strategy in preventing the progression of diabetic complications. In this review, we discuss the most relevant results concerning the neuroprotective effects of flavonoids and vitamins and their underlying mechanisms (Table 1).

## 3. Method of Literature Mining

A computerized search of the “MEDLINE/PubMed” database from 1994 to 2016 for English-language publications was conducted using the following keyword combinations: “bioactive component or food compound or nutrients (baicalein, chrysin, diosmin, epigallocatechin gallate EGCG, hesperidin, kaempferol, luteolin, myricetin, naringenin, proanthocyanidin, puerarin, quercetin, rutin, silibinin, vitamin A, vitamin C, vitamin D and vitamin E)” and “neuronal cell death or neuroprotection or neuronal cell survival, diabetic neuropathy or neuronal function”. The titles and abstracts of the publication hits were subsequently reviewed to select only the papers dealing with the association between bioactive compounds and neuroprotective effects. We included any articles that pertained to the effect of bioactive food compounds on neuroprotection using cell culture and included research on diabetic neuropathy animal models. To evaluate the effects on humans, we searched for relevant reviews, such as cohort/case-control studies, randomized clinical trials and systemic reviews.

## 4. Effect of Flavonoids on Neuronal Cell Death and Dysfunction

Flavonoids are a class of plant and fungus secondary metabolites that are found in fruits, vegetables, grains, roots, stems, flowers, tea and wine. They are divided into flavonols, flavones, flavanols, flavanones, anthocyanidins and isoflavonoids on the basis of their saturation level and opening of the central pyran ring [24]. Flavonoids may play a role in diabetic neuropathy, as shown in several in vitro and in vivo models and some human studies [25]. Flavonoids, such as baicalein, chrysin, diosmin, EGCG, hesperidin, kaempferol, luteolin, myricetin, naringenin, proanthocyanidin, puerarin, quercetin, rutin and silibinin, possess antioxidant, anti-inflammatory and anti-amyloidogenic activities and protect against diabetic neuronal cell death and dysfunction.

### 4.1. Baicalein

Baicalein (5,6,7-trihydroxyflavone), originally isolated from the roots of Scutellaria baicalensis, has been used in traditional Chinese herbal medicine for its antibacterial and antiviral effects since several centuries [89].

Baicalein showed protective effects against amyloid β-(Aβ)-(25–35) and hydroperoxide (H_2_O_2_)-induced neuronal cell injury (rat cortical neurons and human neuroblastoma SH-SY5Y cells) via upregulation of the 12-lipoxygenase and anti-oxidant signaling pathway [26,27]. Li et al. demonstrated that 5 µM baicalein ameliorated lipopolysaccharide (LPS)-induced degeneration of dopaminergic neurons and that the neuroprotective effect of baicalein involved the inhibition of nitric oxide (NO) and free radical release from microglia [28]. Treatment of streptozotocin (STZ)-induced diabetic mice (30 mg/kg/day, intraperitoneally (i.p.) for four weeks) with baicalein significantly reduced diabetic neuropathy, such as motor and sensory nerve conduction velocity deficits, thermal hypoalgesia and tactile allodynia [29]. Although clinical studies are warranted, these results suggest a potential future use of baicalein as a treatment for diabetic neuropathy.

### 4.2. Chrysin

Chrysin (5,7-dihydroxy-2-phenyl-4H-chromen-4-one) is a naturally-occurring flavone, a type of flavonoid, found in honey, fruit and vegetables. Previous studies have demonstrated that chrysin is protective against neuroinflammation and has antioxidant, antidepressant and anti-amyloidogenic effects [31,90].

Chrysin was protective against apoptosis mediated by H_2_O_2_ and the endoplasmic reticulum (ER) stress inducers tunicamycin and staurosporine, as well as attenuated neuronal death. It (4–20 μM) significantly reduced tunicamycin-induced disruption of the mitochondrial membrane potential in SH-SY5Y cells [30,91]. Chrysin also downregulated LPS-induced production of NO, tumor necrosis factor (TNF)-α and interleukin (IL)-1β in primary microglia and the mouse microglial cell line BV-2. The inhibition of nuclear factor kappa kB (NF-κB) and CCAAT/enhancer binding protein (C/EBP)-β and -δ transcription also contributed to the anti-inflammatory effect of chrysin [32,33]. Administration of 30 and 100 mg/kg/day chrysin to STZ-induced diabetic rats for 26 days ameliorated diabetes-associated learning and memory dysfunction. Moreover, chrysin attenuated oxidative stress, as evidenced by increased malondialdehyde (MDA) and decreased catalase (CAT), superoxide mutase (SOD) and glutathione (GSH) in the cerebral cortex and hippocampus [34]. However, chrysin enhanced the formation of Aβ fibril formation, whereas other flavonoids, such as luteolin, quercetin and, myricetin inhibited it [92], suggesting that more studies on the clinical effects of chrysin should be performed.

### 4.3. Diosmin

Diosmin (diosmetin-7-*O*-rutinoside), a natural flavonoid glycoside, is obtained via the dehydrogenation of hesperidin. It is abundant in the pericarp of various citrus fruits and possesses multiple biological activities, including anti-inflammatory, antihyperglycemic, antihyperlipidemic and antioxidant properties [93].

Dholakiya et al. demonstrated that diosmin (1, 3 and 5 μM) treatment, in a dose-dependent manner, reduced the death of PC12 cells (derived from a pheochromocytoma of the rat adrenal medulla) and suppressed LPS-induced TNF-α expression [35]. The effect of diosmin on neuronal cell death induced by other stimuli has not been well reported. In an animal model of diabetes, of Sprague-Dawley (SD) rats fed a high-fat diet and injected with a relatively low concentration of STZ (35 mg/kg), diosmin (50 and 100 mg/kg/day) significantly reduced body weight and glucose levels. Moreover, diosmin, in a dose-dependent manner, improved thermal hyperalgesia, cold allodynia and movement and ameliorated oxidative stress enzyme activity [36]. In STZ-nicotinamide-induced diabetic rats, oral treatment with diosmin (100 mg/kg/day) for 45 days significantly reduced plasma glucose levels and increased non-enzymatic antioxidants (vitamin C and vitamin E) and GSH [37].

A double-blind placebo-controlled study demonstrated that Daflon 500, which is composed of 90% diosmin, reduced HbA1c level, as well as increased glutathione peroxidase activity in type 1 diabetic patients [94].

### 4.4. Epigallocatechin-3-Gallate 

EGCG (((2R,3R)-5,7,-dihydroxy-2(3,4,5-trihydroxyphenyl) chroman-3-yl) 3,4,5-trihydroxybenzoate), which accounts for about one-third of green tea dry mass, is a polyphenolic bioflavonoid derived from a variety of plants, especially green tea. It is responsible for the beneficial, antioxidant effect of the latter. EGCG showed neuroprotective activity against oxidative damage and neurodegeneration [95].

Exposure of hippocampal neurons to Aβ caused marked neuronal injury and increases in MDA levels and caspase activity, while co-treatment with EGCG reduced neuronal apoptosis through scavenging of ROS [38].

Chronic treatment of STZ-induced diabetic rats with EGCG (40 mg/kg/day, orally for seven weeks) reduced hyperalgesia and significantly decreased diabetes-induced thiobarbituric acid reactive substances (TBARS) formation and NO content, as well as reversed the reduction of SOD [39]. Raposo et al. demonstrated that early treatment with EGCG prevented oxidative stress damage (8-hydroxy-2′-deoxyguanosine) and neuronal hyperactivity in the spinal cord and ameliorated behavior related to diabetic neuropathy [40].

### 4.5. Hesperidin

The bioflavonoid hesperidin (3′,5,7-trihydroxy-4′-methoxy-flavanone-7-rhamno glucoside) is a specific flavonoid glycoside that is frequently found in oranges and lemons. It contributes to the intracellular antioxidant defense systems. It acts as a powerful agent against superoxide, singlet oxygen and hydroxyl radicals [96,97,98].

In PC12 cells, hesperidin was protective against Aβ(25–35) by improving mitochondrial function via glycogen synthase kinase (GSK)-3β-mediated voltage-dependent anion channel (VDAC) dephosphorylation [41]. Nones et al. reported that hesperidin treatment decreased the cell death of cortical progenitors obtained from E14 Swiss mice through activation of the PI3K and mitogen-activated protein kinase (MAPK) pathways [42].

Treatment of STZ-induced diabetic rats with hesperidin (50 and 100 mg/kg/day, orally for four weeks) reduced hyperglycemia and restored the decreased nociceptive threshold, motor nerve conduction velocity and sensory nerve conduction velocity. Moreover, hesperidin reduced hyperlipidemia, as well as downregulated free radical generation and pro-inflammatory cytokine production [43]. Pretreatment of STZ-induced mice with hesperidin (100 and 200 mg/kg/day, intracerebroventricular (ICV)) improved memory consolidation processes, possibly through modulation of acetylcholine esterase activity (AChE), a key enzyme that catalyzes the breakdown of the neurotransmitter acetylcholine and choline esters. Moreover, in these mice, hesperidin restored the reduced levels of GSH and elevated the levels of TBARS. Upregulation of inflammatory markers such as NF-κB, inducible NOS and cyclooxygenase-2 in hippocampal neurons was inhibited by hesperidin treatment [44]. The antidepressant effect of hesperidin in STZ-induced diabetic rats was demonstrated after treatment with 25–100 mg/kg/day hesperidin for 21 days; its effect was similar to that of the marketed antidepressant fluoxetine [45]. In vivo and in vitro studies suggest that hesperidin may be a novel neuroprotective compound, indicating the need for clinical studies of the neuroprotective effect of hesperidin.

### 4.6. Kaempferol

Kaempferol (3,5,7-trihydroxy-2-(4-hydroxyphenyl)-4H-1-benzopyran-4-one) is a flavonoid found in several plants, e.g., tea, broccoli, cabbage, kale, beans, endive, leek, tomato, strawberries, grapes and cruciferous vegetables. Kaempferol has a wide range of pharmacological activities, including antioxidant, anti-inflammatory, antidiabetic and neuroprotective effects.

Kaempferol treatment showed efficient neuroprotection against several types of apoptosis- and necrosis-inducing insults. In PC12 cells and primary cortical neurons, kaempferol (25–100 μM) treatment reduced oxidative stress and attenuated H_2_O_2_- and Aβ-induced apoptosis [46,99]. These effects were due to a decrease in the caspase cascade and ROS production [46].

In one study, STZ-induced diabetic mice showed increased levels of TBARS, lipid peroxides and conjugated dienes; kaempferol (100 mg/kg/day, orally) ameliorated hyperglycemia, increased antioxidant status and decreased lipid peroxidation markers [47]. Administration of kaempferol (10–40 mg/kg/day) significantly rescued Aβ-induced impaired performance of diabetic Institute of Cancer Research (ICR) mice in a Y-maze test [99]. Kaempferol-3-*O*-*β*-d-glucopyranoside (a kaempferol derivative) showed stronger inhibitory effects on AGE production than was observed for the positive control (amino guanidine) [100]. Although several studies have reported on the beneficial effects of kaempferol on neuronal cells and diabetic animal models, further studies are needed to confirm these effects in patients.

### 4.7. Luteolin

Luteolin (3′,4′,5,7-tetrahydroxyflavone) is a flavonoid present in many medicinal plants, as well as in some commonly-consumed fruits and vegetables, including green leafy spices, such as parsley, sweet peppers and celery [101]. It shows antioxidant, anti-inflammatory and neuroprotective activities [102,103].

Luteolin (2–50 µM) was neuroprotective against H_2_O_2_-induced cell death in SH-SY5Y cells [91]. Cheng et al. reported that pretreatment with luteolin (1 and 10 μM) concentration-dependently inhibited Aβ (25–35)-induced apoptosis in primary cortical cells, the effect of which was mediated by inhibiting the protein level of extracellular signal-regulated kinase (ERK), c-Jun N-terminal kinase (JNK) and p38 MAPK [48]. In addition to its cytoprotective effect, luteolin is a neurotrophic, as it induces neurite outgrowth, increases the expression of the growth-associated protein-43 (GAP-43), a differentiation marker and activates the ERK-dependent nuclear factor E2-related factor2 (Nrf2) pathway [49]. STZ-induced diabetic rats exhibit neuron damage, cognitive dysfunction and increased oxidative stress and choline esterase (ChE) activity, a marker of cholinergic dysfunction. Chronic treatment with luteolin (50 and 100 mg/kg/day, orally for eight weeks) reduced neuronal injury and improved cognitive performance by attenuating oxidative stress and ChE activity [50]. Treatment of STZ-induced diabetic rats with luteolin (50, 100 and 200 mg/kg/day, i.p. for three weeks) reduced abnormal sensation and improved nerve conduction velocities and nerve flow, and it significantly upregulated Nrf2 and heme oxygenase-1 (HO-1) in diabetic nerves [51], suggesting that the neuroprotective effect involved the Nrf2 pathways. Thus far, no clinical studies of luteolin have been performed, and more studies using diabetic subjects are needed to develop novel therapeutics for the treatment of diabetic neuropathy.

### 4.8. Myricetin

Myricetin (3,5,7,3′,4′,5′-hexahydroxyflavone cannabiscetin) is a natural flavonol found in fruits, vegetables, tea, berries, red wine and medicinal plants [104]. Myricetin has antioxidative and cytoprotective effects, and the results of recent studies suggest that it is a hypoglycemic agent [52,105,106].

Myricetin has anti-aldose reductase activity and may play an important role in the development of diabetic neuropathy [107]. Myricetin (300 nM) potentially reduced Aβ(1–42)-induced cell injury in rat cortical neurons [52]. Moreover, myricetin markedly inhibited the cross-linking formation of AGE in collagen incubated with glucose-treated cells compared to the inhibition by quercetin, rutin, catechin and kaempferol, suggesting that myricetin might be a highly potent inhibitor of AGEs [108]. Although myricetin showed promising antihyperglycemic and antidyslipidemic effects in some studies [109,110], its potential antineuropathic effect requires a detailed study using animal models of diabetes.

### 4.9. Naringenin

Naringenin (4′,5,7-trihydroxyflavanone) is a biological active molecule found in citrus fruits, such as grapefruits and oranges, and tomatoes [111]. Biological activities, such as antioxidant, antitumor and anti-inflammatory effects, as well as activation of peroxisome proliferator-activator receptors (PPARs), have been observed [112]. Furthermore, decreased diabetic neuropathy has been reported, as well [113,114].

Treatment of glial cells with naringenin protected against LPS-/interferon (IFN)-induced neuroinflammatory injury via downregulation of p38 MAPK phosphorylation and signal transducer and activator of transcription-1 (STAT-1) [53]. In contrast, suppressor of cytokine signaling (SOCS)-3 was upregulated by naringenin treatment [54]. Further, treatment of STZ-induced diabetic rats with 50 and 100 mg/kg/day naringenin (orally for eight weeks) reversed chemical and thermal hyperalgesia and decreased hyperglycemia, as well as restored SOD activity [55]. Al-Rejaje et al. demonstrated that naringenin treatment (25 and 50 mg/kg/day) significantly decreased the level of oxidative stress biomarkers and increased NGF in sciatic nerves of STZ-induced diabetic rats [56].

### 4.10. Proanthocyanidin

Proanthocyanidin ((3R)-2-(3,5-dihydroxy-4-methoxyphenyl)-8-((2R,3R,4R)-3,5,7 trihydroxy-2-(4-hydroxyphenyl)-3,4-dihydro-2H-chromen-4-yl)-3,4-dihydro-2H-chromene-3,5, 7-triol) is known as a condensed tannin, a member of a specific group of polyphenolic compounds derived from grape seeds. It has been reported to exhibit strong antioxidant activity [115].

Pretreatment with proanthocyanidin (50 mg/L) decreased H_2_O_2_-induced reduction of the viability of mouse primary glial cells and PC12 cells [57,58]. The attenuating effect of proanthocyanidin on diabetic neuropathy has been investigated in various animal models of diabetes. Proanthocyanidin (250 mg/kg/day for 24 weeks) significantly increased motor nerve conductive velocity, mechanical hyperalgesia and SOD activity and decreased serum glucose, AGEs and MDA in STZ-induced diabetic rats [59]. STZ-, high-carbohydrate- and high-fat diet-induced diabetic SD rats fed proanthocyanidins (250 mg/kg/day) exhibited significantly decreased LDL levels and increased nerve conduction velocity compared to those in untreated controls. In addition, proanthocyanidin and its metabolites catechin and epicatechin reduced cell injury and downregulated the expression level of ER stress proteins in tunicamycin-treated sciatic nerves and rat Schwann cells (RSC cells) [60]. Intracerebroventricular (ICV) injection of Aβ(25–35) in C57BL/6 mice impaired learning and memory, which were attenuated after oral administration of proanthocyanidin (50 and 100 mg/kg/day) as a result of decreased neuronal apoptosis in the hippocampus and increased synaptic density [61]. Natella et al. demonstrated that administration of 300 mg/day of proanthocyanidin-rich grape seed extracts minimized postprandial oxidative stress by increasing plasma antioxidant levels in healthy men (25–40 years old) [116]. However, as the sample size was small, a large-scale study in humans is necessary.

### 4.11. Puerarin

Puerarin (daidzein-8-C-glucoside 7,4′-dihydroxy-8-C-glucosylisoflavone), a naturally-occurring isoflavone C-glycoside, was isolated from Pueraria lobata. Puerarin is used in the treatment of several diseases because of its rapid absorption from the intestine, distribution to the brain via specific transport pathways and low toxicity [117].

Puerarin reduced Aβ- and H_2_O_2_-induced cell injury [62,118], increased AKT/PI3K [64] and decreased caspase-3 and caspase-9 activity [63]. Further, its neuroprotective effect in vitro involved GSK-mediated Nrf2 activation [65]. Intrathecal administration of puerarin (4–100 nM/day for seven days) to STZ-induced diabetic neuropathic pain rats inhibited the mechanical and thermal nociceptive response and also reduced the upregulated levels of NF-κB and the inflammatory cytokines IL-6, IL-1β and TNF-α in the spinal cord [66]. The results of different randomized controlled studies (22 studies with 1664 participants) were analyzed by Wu et al., showing that puerarin could improve the total effective rate, restore the diabetes-induced decrease in nerve conduction velocity and improve the hemorheology index [119].

### 4.12. Quercetin

Quercetin (3,3′,4′,5′,7-pentahydroxy flavone) is a flavonoid that is naturally present in various foods, such as onions, apples, broccoli, tea and red wine [120]. Quercetin has several beneficial pharmacological properties, such as anticarcinogenic, anti-inflammatory and antioxidant activity, as well as anti-diabetic effects [121].

In general, the addition of quercetin protects against H_2_O_2_- and glucose-induced toxicity and promotes neuronal cell proliferation. Exposure of PC12 cells to quercetin alone (10–30 μM), however, caused cell death and enhanced H_2_O_2_-induced (0.1 mM) cell death [122]. In SH-SY5Y cells, on the other hand, quercetin inhibited H_2_O_2_-induced (0.5 mM) cell death and inhibited Krüppel-like factor 4 (KLF4), which is a zinc finger transcription factor playing a role in cell proliferation, differentiation and apoptosis [67]. Quercetin was protective against high glucose-induced injury (45 mM for 24 h or 125 mM for 72 h) in dorsal root ganglion (DRG) neurons, primary Schwann cells and RSC cells by activating Nrf-2/HO-1 and inhibiting NF-κB [68]. Qu et al. reported that quercetin could alleviate high glucose-induced damage to Schwann cells by increasing autophagy and proliferative activity. Low expression of beclin-1 and light chain 3 (LC3), the molecular markers for autophagy, caused by high glucose treatment was rescued by quercetin treatment [69]. The growth-promoting effect on Schwann cells was also observed with 0.1, 1 and 10 µg/mL quercetin [70].

Several studies have shown that quercetin can exert neuroprotective effects in vivo. A four-week treatment course of quercetin (100 mg/kg/day, orally) ameliorated thermal hyperalgesia in an STZ-induced diabetic neuropathic pain model by inhibiting PKC [123]. Administration of a high dose of quercetin (200 mg/kg/day) to STZ-induced diabetic rats did not affect glucose levels, but prevented neuronal loss and increased the number of glial cells compared to control mice [124]. In mice fed a high-fat diet, quercetin (0.5–50 mg/kg/day for 13 weeks) was protective against oxidative stress and improved cognitive function [71]. SD rats that were administered quercetin (0.2 pg/day −0.2 µg/day) for eight weeks exhibited an increase in myelinated axons, suggesting that quercetin might be an addition to or replacement of neurotrophic factors to promote nerve regeneration [70].

Although several studies have assessed the anti-diabetic neuropathic activity in in vivo models, only a few clinical studies have assessed the effects of quercetin in humans. For example, Valensi et al. showed in a randomized, placebo-controlled, double blind trial involving diabetic neuropathy patients that application of QR-333, a topical compound containing quercetin, three times daily for four weeks safely induced relief from symptoms (e.g., foot pain, irritation and sensitivity) related to diabetic neuropathy and improved quality of life [125].

### 4.13. Rutin

Rutin (3,3′,4′,5,7-pentahydroxyflavone-3-rhamnoglucoside) is one of the most common native flavonoids present in food, including onions, apples, tea and red wine [126]. Rutin exhibits both antidiabetic and anti-inflammatory properties [127].

In neuronal cells, rutin protects against various neurotoxicities induced by the prion peptide [128], ethanol [129], glutamate [130] and dexamethasone [131]. Nevertheless, its protective effects against high glucose-, H_2_O_2_- and Aβ-induced toxicities have not been reported.

Oral administration of rutin (100 mg/kg/day for 45 days) to STZ-induced diabetic rats significantly decreased fasting glucose and glycosylated hemoglobin and increased insulin and C-peptide. Moreover, rutin-treated rats showed antioxidant activity, as demonstrated by significant decreases in TBARS and lipid hydroperoxides and increases in non-enzymatic antioxidants [72]. Recently, Tian et al. demonstrated that rutin (5, 25 and 50 mg/kg/day, i.p. for two weeks) reduced mechanical hyperalgesia, heat hyperalgesia and cold allodynia in STZ-induced diabetic rats, significantly increased Na^+^, K^+^-ATPase activity in diabetic nerves and decreased caspase-3 expression in DRG neurons. In addition, rutin significantly decreased the plasma glucose levels and attenuated oxidative stress via upregulation of the Nrf2 signaling pathway [73].

A combination therapy of rutin (50 mg/kg/day) and silymarin in STZ-induced diabetic rats for six weeks reduced plasma glucose levels and significantly increased SOD, CAT and GST levels in the sciatic nerve of diabetic rats. Moreover, rutin reduced hyperalgesia, analgesia and improved motor coordination in diabetic rats [132].

### 4.14. Silibinin

Silibinin ((2*R*,3*R*)-3,5,7-trihydroxy-2-((2*R*,3*R*)-3-(4-hydroxy-3-methoxyphenyl)-2- (hydroxymethyl)-2,3-dihydrobenzo[*b*][1,4]dioxin-6-yl)chroman-4-one) is a flavonoid and the major active constituent of silymarin (known as milk thistle seed extract). It has been proposed to have anticancer, antioxidant, anti-inflammatory and neuroprotective effects.

The neuroprotective activity of silibinin was observed in experimental models, such as 1-methyl-4-phenylpyridine and aluminum-induced neurotoxicity [133,134,135]. Under conditions of oxidative stress induced by Aβ, silibinin prevented H_2_O_2_ production in SH-SY5Y cells [74]. Recently, Wang et al. demonstrated that silibinin promoted neuron viability upon H_2_O_2_ treatment in cortical neuron cells and reduced cerebral ischemia reperfusion injury in mouse [75]. Administration of silibinin (20 mg/kg/day, i.p. for four weeks) reduced DNA damage and oxidative stress in a brain of db/db mice, and the heme oxygenase-1 pathway was involved in the neuroprotective effect [76]. These results indicate that silibinin may be a novel therapeutic agent for the treatment of neuronal cell death in diabetes.

## 5. Effect of Vitamins on Neuronal Cell Death and Dysfunction

Dietary vitamins, such as vitamins A, C, D and E, have a number of biological activities, including immunostimulatory effects, prevention of ROS-induced DNA damage and neuritogenic activities. These properties are known to alleviate diabetes-induced neuronal cell death and dysfunction in in vitro, in vivo and clinical studies.

### 5.1. Vitamin A

Vitamin A is an essential dietary nutrient required for normal growth, reproduction and vision. Root vegetables and greens, such as squash, carrots, pumpkins and beet greens, are rich sources of vitamin A. Retinoic acid, a physiologically-active retinoid synthesized from vitamin A, regulates neuronal differentiation during embryonic development and is required for the maintenance of plasticity in differentiated neurons. Retinoic acid (300 nM) induced neuronal differentiation as measured by neurite outgrowth of cortical neurons from rat embryos (Embryonic Day 16) by activating Rac1 [77]. De Bittencourt Pasquali et al. demonstrated that the glycolytic and antioxidant pathways were involved in the differentiation of SH-SY5Y cells by retinoic acid [78]. Treatment of diabetic mice with 20 mg/kg/day retinoic acid for 60 days significantly increased nerve and serum NGF levels, nerve regeneration (myelinated axons and Schwann cells) and nerve sensitivity [136,137]. These results suggest that retinoic acid is a potential therapeutic agent for the treatment of nerve degeneration and dysfunction in diabetes.

### 5.2. Vitamin C

Vitamin C (ascorbic acid), a water-soluble anti-oxidant vitamin, is widely distributed in fresh fruits and vegetables and plays an important role in protecting against free radical-induced damage. Neurons in the CNS contain the highest ascorbic acid concentrations [138], and intracellular ascorbate plays several functions, including antioxidant activity, peptide amidation, myelin formation, synaptic potentiation and neuroprotection against toxicities, such as ethanol, methamphetamine and lead [139,140,141,142].

Ascorbate treatment (100 μM) protects Aβ (25–35)-induced toxicity in SH-SY5Y cells [79] and high glucose-induced apoptosis in human brain pericytes [80]. However, the studies investigating the effect of vitamin C in diabetic animal models and diabetic patients have not been well investigated. Therefore, more research is needed to ascertain the effect of vitamin C in diabetic neuropathy.

### 5.3. Vitamin D

The various forms of vitamin D comprise a group of essential steroid hormones that are synthesized under exposure to sunlight and are also absorbed from foods. Foods containing high levels of vitamin D are fatty fish and their liver-derived oils, eggs and fortified foods. Although the role of vitamin D in neuronal physiology remains unclear, some studies have found a role for vitamin D in neuronal cells. 

NGF, neurotrophin and glial cell line-derived neurotrophic factor (GDNF) synthesis were upregulated by 1,25(OH)_2_D_3_, the active form of vitamin D, in neuronal cells [81,82,83]. A vitamin D3 derivative (CB1093, 1(S),3(R)-dihydroxy-20(R)-(1-ethoxy-5-ethyl-5-hydroxy-2-heptyn-1-yl) -9,10-seco-pregna-5(Z),7(E),10(19)-triene; 0.3 and 1 μg/kg/day) also caused a dose-dependent increase in NGF production in the sciatic nerve of diabetic rats; however, it did not change glucose levels [84].

In a study in humans, it was reported that vitamin D deficiency in diabetes was associated with symptoms of diabetic neuropathy, e.g., pain, loss of feeling and tingling in the hands and/or feet [143]. In a non-randomized study of vitamin D supplementation comprising 51 type 1 diabetes subjects with painful diabetic neuropathy, 50% of the patients had a decrease in pain scores [144].

### 5.4. Vitamin E

Vitamin E belongs to a group of fat-soluble vitamins and is found predominantly in oily plants. Nuts, seeds and oils are rich sources of vitamin E. Vitamin E is a potent antioxidant with anti-inflammatory properties [145]. Several lines of evidence have suggested that among the different forms of vitamin E, α-tocopherol has beneficial effects against diabetic neurocytotoxicity.

In in vitro studies, vitamin E protected neurons against the toxicity of Aβ, high glucose and H_2_O_2_. At concentrations as low as 100 nM, α-tocopherol was protective against oxidative cell death caused by Aβ, H_2_O_2_ and the amino acid glutamate in HT22 cells (mouse hippocampal cells) and rat cerebellar granule neurons. Moreover, vitamin E increased the activity of NF-*κ*B, which is involved in the control of nerve cell survival [85]. A high dose of α-tocopherol (1 mM) reduced H_2_O_2_-induced oxidative stress. In addition, heat shock protein 60 and vimentin, two anti-apoptotic proteins, were not oxidized in the presence of α-tocopherol (1 mM), which thus prevented cellular apoptosis [86]. In cultured embryo tissues, high glucose treatment inhibited Pax-3 expression, which is required for neural tube closure and neural tube defects, and this effect was blocked by α-tocopherol [87]. Several studies have shown that diabetic rats fed a vitamin E-supplemented diet demonstrated improved nerve conduction in sensory and motor nerves [146,147]. Further, reactive astrocytosis, which is associated with lipid peroxidation, was prevented in STZ-induced diabetic rats fed a vitamin E-supplemented diet [88]. Vitamin E plays an important role in diabetic patients, as well. The plasma vitamin E: Lipid ratio was lower in diabetics with neuropathy than that in controls [8]. In a study of type 2 diabetes mellitus patients, 88% of the study population reported relief from neuropathic pain after a 400-mg vitamin E dose in combination with evening primrose (500–1000 mg/day) [148].

## 6. Conclusions

As the prevalence of diabetes and its complications continue to increase rapidly, there is an increasing need for the development of safe and effective functional bioactive compounds with antidiabetic effects. The discussed flavonoids and vitamins regulate neuronal cell survival and function by promoting proliferation and neurite outgrowth and reducing apoptosis, inflammation and oxidative stress. Determining the molecular mechanisms underlying the amelioration of hyperglycemia, hyperlipidemia and impaired insulin signaling may aid the development of new drugs for diabetic neuropathy. Moreover, such a neuroprotective effect may also have significant effects on neurodegenerative disorders, such as Alzheimer’s disease and Parkinson’s disease. The bioactive compounds discussed in this review may prove to be excellent alternative therapeutic strategies, especially given the positive outcomes of clinical studies of some of these bioactive compounds, such as diosmin, EGCG, proanthocyanidin, puerarin, quercetin, vitamin D and vitamin E. However, further clinical trials of the effect of these bioactive compounds are warranted to provide a stronger foundation for their potential future therapeutic applications.

## Figures and Tables

**Table 1:** Representative flavonoids and vitamins showing neuroprotective effects under diabetic conditions.

Bioactive Compounds	Models	Effects	Specific Mechanisms of Action	Reference
Flavonoids (Subclass)				
Baicalein (flavones)	Primary rat cortical neurons	↓Aβ-induced cell death	↓12-lipoxygenase	[26]
	SH-SY5Y cells	↓H_2_O_2_-induced cell death	↓oxidative stress	[27]
	Primary dopaminergic neurons	↓LPS-induced cell injury	↓NO, free radicals	[28]
	STZ-induced diabetic mice	↑nerve conductive velocity	↓oxidative-nitrosative stress and p38 MAPK	[29]
Chrysin (flavones)	SH-SY5Y cells	↓ER stress cell death	↑mitochondrial membrane potential	[30]
	Primary microglia/microglia cell line	↓LPS-induced NO, TNF-α and IL-1β	↓JNK, NF-κB and CEBPβ	[31,32,33]
	STZ-induced diabetic rats	↑learning and memory function	↑CAT, SOD, GSH/ ↓MDA	[34]
Diosmin (flavones)	PC12 cells	↓LPS-induced apoptosis	↓TNF-α	[35]
	High-fat diet-/STZ-induced diabetic mice	↓glucose level and body weight ↑nerve function	↓oxidative stress enzyme activity	[36]
	STZ-/nicotinamide-induced diabetic mice	↓glucose level	↑antioxidants (vitamin c, vitamin E) and GSH	[37]
EGCG (flavanol)	Hippocampal neuronal cells	↓Aβ-induced injury	↑MDA and caspase activity	[38]
	STZ-induced diabetic rats	↓hyperalgesia	↓TBARS and NO ↑SOD	[39]
	STZ-induced diabetic rats	↓hypersensitivity	↓oxidative stress damage	[40]
Hesperidin (flavanones)	PC12 cells	↓Aβ-induced apoptosis	↑GSK3β-mediated VDAC	[41]
	Cortical progenitors	↓cell death	↑PI3K and MAPK	[42]
	STZ-induced diabetic rats	↓hyperglycemia and hyperlipidemia ↑nerve function	↓free radical generation and proinflammatory cytokines	[43]
	STZ-induced diabetic mice	↑nerve function	↑AchE and GSH ↓TBARS, NF-κB, iNOS and COX-2	[44,45]
Kaempferol (flavonols)	HT22 cells	↓H_2_O_2_-induced apoptosis	↓ROS production	[46]
	STZ-induced diabetic mice	↓glucose level	↓lipid peroxidation	[47]
Luteolin (flavones)	Primary cortical neurons	↓Aβ-induced cell death	↓ERK, JNK, p38 MAPK	[48]
	SH-SY5Y cells	↑neurite outgrowth	↑ERK-dependent Nrf2 pathway	[49]
	STZ-induced diabetic rats	↓neuronal injury ↑cognitive performance	↓oxidative stress and ChE activity	[50]
	STZ-induced diabetic rats	↑nerve conduction	↑Nrf2 and HO-1	[51]
Myricetin (flavonols)	Rat cortical neurons	↓Aβ-induced cell injury	↓AGE	[52]
Naringenin (flavones)	Primary microglial cells	↓LPS-induced cytokine release	↓p38 MAPK, STAT-1 ↑SOCS3	[53,54]
	STZ-induced diabetic rats	↓glucose level ↑NGF, IGF	↑SOD, CAT, GPx	[55,56]
Proanthocyanidin (flavanols)	Mouse primary microglia cells and PC12	↓H_2_O_2_-induced cell death	↓lactate dehydrogenase	[57,58]
	STZ-induced diabetic rats	↓glucose level ↑nerve conductive velocity	↑SOD, ↓AGE and MDA	[59]
	STZ-/high carbohydrate-/high-fat diet-induced diabetic rats	↓LDL ↑nerve conductive velocity	↓ER stress protein	[60]
	Aβ-induced diabetic mice	↓neuronal apoptosis ↑synaptic density	↑antioxidant level	[61]
Puerarin (isoflavones)	PC12 cells	↓H_2_O_2_-induced cell death	↑caspase-3, caspase-9 ↑SOD, GSHAKT/PI3K	[62,63]
	PC12 cells	↓Aβ-induced cell death	↑AKT/PI3K	[64]
	Primary rat hippocampal neurons	↓Aβ-induced oxidative stress	↑Nrf-2/HO-1	[65]
	STZ-induced diabetic rats	↓pain sensitivity	↓inflammatory cytokines	[66]
Quercetin (flavonols)	SH-SY5Y cells	↓H_2_O_2_-induced cell death	↓KLF4	[67]
	Dorsal root ganglion cells, primary Schwann cells and RSC96 cells	↓high-glucose injury	↑Nrf-2/HO-1, ↓NF-kB	[68,69]
	Schwann cells	↑growth ↓high glucose-induced damage	↑autophagy	[69,70]
	High-fat diet-induced diabetic mice	↑cognitive function	↓oxidative stress enzyme activity	[71]
Rutin (flavonols)	STZ-induced diabetic rats	↓glucose level	↓TBARS and lipid hydroperoxides	[72]
	STZ-induced diabetic rats	↓glucose level ↑nerve function	↑Nrf-2	[73]
Silibinin	SH-SY5Y cells	↓Aβ induced cytotoxicity	↓oxidative stress	[74]
	Mouse cortical neurons	↓H_2_O_2_-induced cell death	↓beclin-1, LC3-II expression	[75]
	db/db mice	↓oxidative stress ↑DNA protection	↑HO-1	[76]
Vitamins				
Vitamin A	Rat embryonic cortical neurons	↑neurite outgrowth	↑RAC1	[77]
	SH-SY5Y cells	↑neuronal differentiation	↑glycolytic pathway and antioxidant pathway	[78]
Vitamin C	SH-SY5Y cells	↓Aβ induced cytotoxicity	↓oxidative stress	[79]
	Human brain pericytes	↓high glucose induced apoptosis	↓advanced glycation end production	[80]
Vitamin D	Primary astrocytes/C6 glioma cells	↑NGF, GDNF and neurotrophin	-	[81,82,83]
	STZ-induced diabetic rats	no changes in glucose levels	↑NGF level	[84]
Vitamin E	HT22 cells/rat cerebellar granule neurons	↓Aβ- and H_2_O_2_-induced cell death	↑NF-κB activity ↓HSP60 and vimentin	[85,86]
	*Ex vivo* embryo tissues	↓high glucose-induced neuronal tube defect	↑Pax-3 expression	[87]
	STZ-induced diabetic rats	↓reactive astrocytosis	↓lipid peroxidation	[88]
